# Household-income trajectories and mental health inequalities in Germany before, during, and after the COVID-19 pandemic: a quasi-experimental panel study

**DOI:** 10.1186/s12939-025-02507-1

**Published:** 2025-05-19

**Authors:** Ibrahim Demirer, Heike Krüger, Timo-Kolja Pförtner

**Affiliations:** 1 Department for Psychosomatic Medicine and Psychotherapy, Medical Faculty, Center for Health Communication and Health Services Research University Hospital Bonn University of Bonn, Bonn, Germany; 2 Department of Research Methods, Faculty of Human Sciences, University of Cologne, Cologne, Germany; 3 Institute of Sociology, Faculty of Arts and Humanities, RWTH Aachen University, Aachen, Germany; 4 Institute of Sociology and Social Psychology, Faculty of Management, Economics and Social Sciences, University of Cologne, Cologne, Germany

## Abstract

**Background:**

The COVID-19 pandemic disrupted progress toward achieving the Sustainable Development Goals (SDGs), particularly SDG 10 (Reduced Inequalities) and SDG 3 (Good Health and Well-Being). In Germany, labor market volatility, compounded by record inflation, widened social inequalities and contributed to a gradient in mental health. This study examines the relationship between household income and mental health before, during, and after the pandemic in the German working population, addressing whether mental health burdens persisted post-pandemic.

**Methods:**

Using the German Socioeconomic Panel (v39.0), we applied a quasi-experimental design employing a ‘placebo control’ approach to obtain difference-in-difference (DiD) estimates. For this purpose, we created an ‘intervention’ sample consisting of respondents exposed to the COVID-19 pandemic (*N* = 8,340, 2018–2022) and a ‘placebo control’ sample, consisting of respondents not exposed to the COVID-19 pandemic (*N* = 11,869, 2014–2018), designed to mimic the intervention sample. Sequence analysis identified six typical household income trajectories (S1–S6): high, regular, fluctuating-I-II, low and unemployed. We used estimation methods to assess the mental health impacts of these trajectories during and post-pandemic, stratified by gender.

**Results:**

The results confirmed a strong social gradient in mental health tied to household income. For males, the COVID-19 pandemic caused a mental health decline of ~ ¼ standard deviation for trajectories reflecting regular (S2), fluctuating (S3), and low household income (S5) (e.g., S3-DiD = -2.043^**^), while those in high household income or unemployed trajectories were unaffected. Females experienced a more generalized mental health decline across all trajectories. Post-pandemic, mental health showed signs of recovery but did not fully return to pre-pandemic levels.

**Conclusions:**

These findings revealed that regular and fluctuating household income trajectories (S2–S5) are particularly vulnerable to mental health impacts during crises. Females are disproportionately affected, highlighting the need for targeted public health interventions. Strengthening institutional supports, such as childcare, and addressing gender disparities can help build resilience and advance progress toward the SDGs.

**Supplementary Information:**

The online version contains supplementary material available at 10.1186/s12939-025-02507-1.

## Background

Previous research consistently finds that household income trajectories are associated with the prevalence of lifetime mental health [1]. This association appears to be primarily unidirectional, with evidence suggesting that reductions in household income causally contribute to deteriorations in mental health [2]. Consequently, increases in income above the poverty threshold are positively associated with improvements in mental health [3]. Several theoretical frameworks offer explanations for the robust link between income and mental health. According to conservation of resources theory [4], individuals with low income face a double jeopardy: First, through the lack and loss of resources, and the threat thereof producing stress. Second, by coping with stressors and the resulting inability of resource consumption instead of the desired resource accumulation. Stress-exposure theory further posits that individuals with fewer resources are particularly vulnerable to stressors, as they are less able to buffer their effects due to limited access to financial means and healthcare [5]. Capability approaches argue that income trajectories not only affect health through material deprivation, but also by undermining autonomy, social participation, and perceived agency [6]. From a life course perspective, economic disadvantage tends to accumulate over time, contributing to the widening of health inequalities as individuals age [7, 8]. The importance of accounting for these accumulation processes is empirically recognized by previous research through utilization of income trajectory measures instead of static income measures [7, 9–11].

Gender differences are evident in the income – mental health association, with male’s mental health being more vulnerable in low income trajectories [10], and an early start to the accumulation of disadvantage [12, 13]. For females, however, the interplay between labor-market participation and family work is important. Studies have found early parenthood and low labor-market ties to associate with distress in later life [14–16]. Especially in Germany, where the labor market continues to reflect a modified male breadwinner model [17, 18], mental health inequalities intersect strongly with gender and employment [19]. In low-income trajectories females are disproportionately overrepresented, and even in dual-earner couples, females contribute less to the household income [20]. As a result, forms of (voluntary) unsecure and low income employment are more common among females, who may nevertheless reside in economically secure households [21, 22]. On the opposite, males tend to achieve a higher income and experience a stronger increase in income over the life course [23], often due to persistent structural barriers such as unequal division of care responsibilities, insufficient institutional support, and entrenched gender norms [24]. Role strain theory predicts that individuals may experience role conflict when the fulfillment of competing social roles, such as caregiving and employment, is in conflict. This, in turn, may contribute to negative mental health outcomes [25, 26]. Simultaneously, for females, mental health may be more reactive to acute external stressors [27], due to the exposure of multiple simultaneous stress domains and social responsibility burdens. Their greater reliance on social support networks as a coping strategy [28], while generally protective, became a particular risk factor during the pandemic, when social distancing measures significantly disrupted access to such resources.

Concerning the COVID-19 pandemic, economic resources are particularly important in times of crisis, and previous research on crises has shown that vulnerable labor-market participants are more likely to suffer adverse mental health outcomes [29, 30]. The COVID-19 pandemic came as an exogenous event on top of the pre-existing social gradient in mental health, and its accompanying economic hardship due to instability in the financial and labor markets was found to increase economic worries for most labor market participants [31, 32]. Simultaneously, the COVID-19 pandemic placed an additional burden on mental health, as evidenced by the widespread lockdowns and social distancing measures that significantly reduced social interactions, leading to feelings of loneliness and isolation [33]. As social connections are essential for mental well-being, their abrupt disruption led to increased levels of anxiety, depression, and emotional distress [34–36], as well as mental health care utilization [37, 38]. Against this background, existing social inequalities seem to have increased during the COVID-19 pandemic, and, thereby, the gradient in mental health [39–41]. These processes appear to have thwarted the Sustainable Development Goals (SDGs) health and wellbeing (SDG3) and less social inequality (SDG10).

However, research has yet to understand the post-pandemic behaviour of the association between economic resource trajectories and mental health. Specifically, if adverse mental health effects during the pandemic are found – for whom were they more elevated? – Secondly, it is crucial to ascertain whether the found differences recover one-year after the COVID-19 pandemic. Addressing these questions is important for reducing the widening social inequality in mental health. Specifically, this study investigates the mental health gradient across different household income trajectories, highlighting how these trajectories intersect with mental health and gender in a pre-, per-, and post-pandemic context. Given the complexities of investigating such longitudinal processes throughout different periods with observational data, this study also outlines and addresses the involved methodological challenges.

## Methods

### Data strategy

**Fig. 1:**
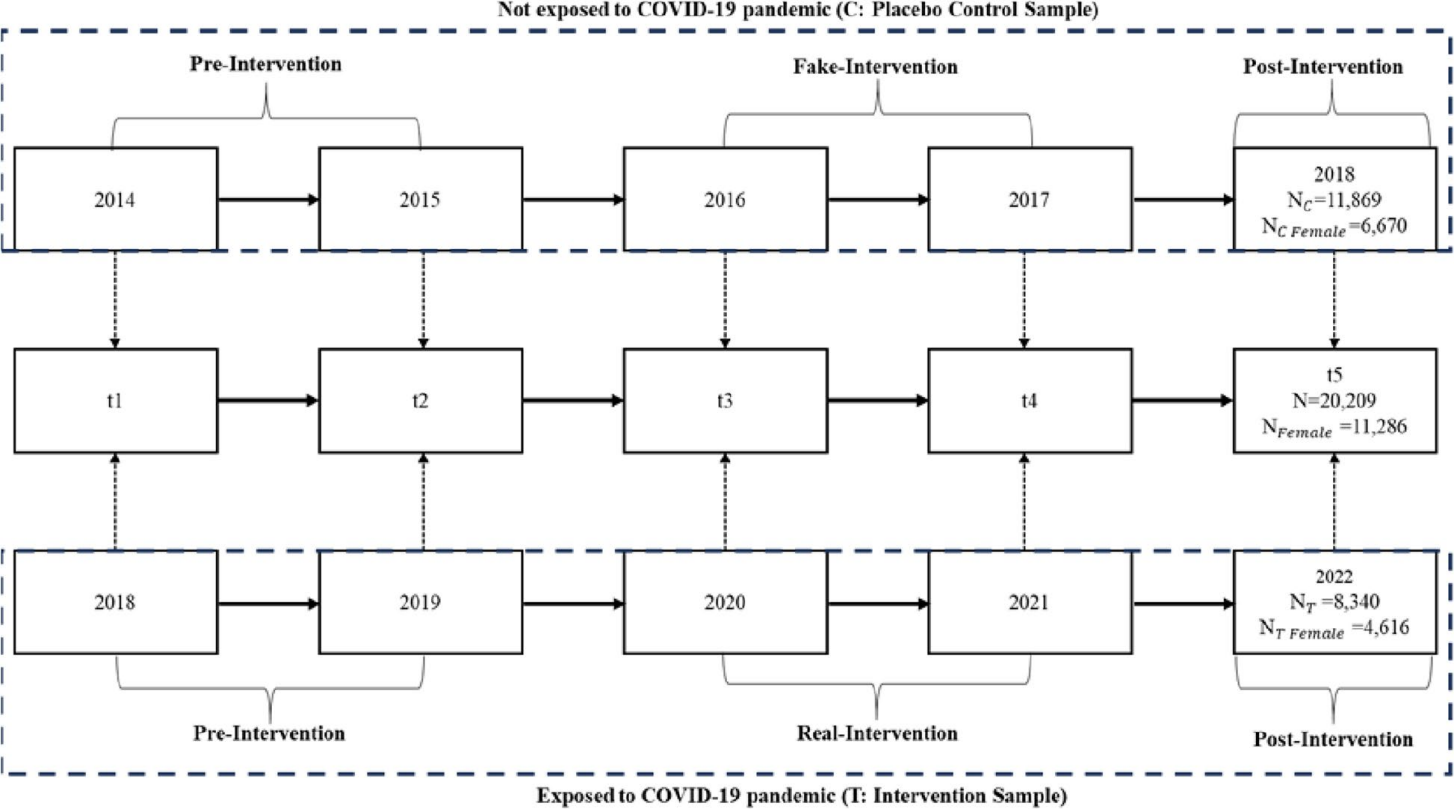
Flowchart of data division and period classification for placebo control and intervention sample using the GSOEP

Starting in 1984 as an annual survey, the German Socioeconomic Panel (GSOEP) is the largest ongoing panel study in Germany, with the latest wave consisting of data collected in 2022 [42]. We analyzed data from the annual waves of the GSOEP, covering the years 2014 to 2022. With these data, we first applied sample division and then period classification. The sample division grouped the data into two samples, with the ‘intervention sample’ (T), compromising respondents exposed to the COVID-19 pandemic and consisting of five waves between 2018 to 2022 (T_t1_-T_t5_) and the ‘placebo control sample’ (C) consisting of five waves from 2014 to 2018 (C_t1_-C_t5_) with respondents unexposed to the COVID-19 pandemic. Furthermore, the samples were restricted to individuals with valid entries on the main analysis variables and who were not older than 60 at t1 of the respective sample. This was done to ensure that the household income trajectories were not disrupted by retirement entries. These samples were then matched across the five time points (t1-t5), resulting in a single panel dataset of 20,417 individuals, of whom N_T_ = 8,444 and N_C_ = 11,973. In the combined panel sample, we then classified the periods as following: t1-t2 (pre-COVID), t3-t4 (per-COVID, t5 (post-COVID). The sampling division and period classification strategy is visualized in Fig. 1.

This approach utilizes the quasi-experimental assignment process of the COVID-19 pandemic for the intervention sample in a pre-, per-, and post-COVID-19 period, and mimics this assignment process for the control sample [43, 44]. In detail, the control sample was observed entirely prior to the pandemic, with the corresponding time points t3-t4 (per) and t5 (post) treated as “fake” intervention periods to mirror the timeline of the intervention sample. The use of a placebo control test ensures that the parallel-trend assumption, which is a prerequisite for the DiD estimation, holds true [45]. This is the case when there is no effect for the placebo control group. Furthermore, our approach enables the comparison of any pre-intervention discrepancies between the control and intervention samples [46]. Any differences between the pre-intervention episodes of the control and intervention samples should be eliminated.

### Exposure variable: net-household equivalised income

As a measure of available economic resources, we utilized the Net-Household Equivalised Income (HH-EI). While personal income captures only individual earnings, HH-EI accounts for the combined financial contributions and shared burdens of all household members [47], thereby more accurately representing the economic resources that may affect mental health. The annual information on HH-EI was combined with the information on employment status to generate the HH-EI variable (element variable). This categorical variable was coded as 1 ‘high HH-EI’ if the respondent was in the top 10 th percent of the net-household-equivalence-income distribution. Respondents below that distribution but above 60% median of the net-household-equivalence-income were coded as 2 ‘regular HH-EI. In accordance with the OECD definition of relative poverty, respondents whose income fell below the 60% median income were coded as 3 ‘low HH-EI’. Lastly, as a somewhat residual category, respondents without employment received the value 4 ‘unemployed’. With this categorization of the element variable, we try to capture relevant differences in HH-EI levels, while taking into account unemployment as an extra category rather than, e.g., subsuming it under low HH-EI.

### Outcome variable: mental health

The GSOEP surveys mental health every two years using the Mental Component Summary (MCS), a measure included in the Short Form-12 Health Survey (SF-12v2). The SF-12v2, derived from the 36-item SF-36v2, is a multidimensional tool for evaluating health-related quality of life, compromising 12 items. The MCS scores range from 0 to 100 (T-score) with a standard deviation (SD) of 10, where higher scores indicate better mental health [48]. Data on the MCS were collected at three time points: t1 (pre-COVID-19), t3 (during COVID-19), and t5 (post-COVID-19).

### Covariates: sample selectivity and confounding

In addition to the HH-EI, age, mental health and gender, we included additional covariates with the objective of reducing the selectivity of participation in the survey during the period of the pandemic, and to control for the confounding effects between HH-EI trajectory and mental health. Most importantly, all analyses were stratified for gender (binary) and then adjusted for socio-demographics, consisting of: age (quadratic and cubic), migration background (yes/no), highest educational degree (Casmin three categories), ISEI socioeconomic status (continuous) and East vs. West German residency. Studies have demonstrated age to be non-linear associated with employment type and health [49], and migration background to be negatively associated with occupational health and socioeconomic status [50]. Local labor markets not only affect the HH-EI trajectories but eventually mental health as well [51], which, in Germany, is still marked by the West vs. East differences [52]. Similarly, these factors appear to influence the participation rates observed in panel studies during the period of the COVID-19 pandemic [53]. The GSOEP data set was also subjected to adjustments pertaining to the time elapsed since a given respondent had participated in the study, as well as the region and interview month of the survey.

Furthermore, we adjusted for social dimensions, including the family status (categorical: married, partnership, no partnership, divorced/widowed) and the number of children in the household (continuous), as the COVID-19 pandemic appears to increase the mental health disparities among families [54]. Concerning, partnerships, we have also included information on the employment status of partners (binary: partner employed; partner unemployed) as a potential confounder, assuming that it contributes to voluntary unemployment in cases of high HH-EI, thus influencing the association between the HH-EI trajectory and mental health [21]. Finally, physical health (physical component score SF-12v2, PCS) was defined as a confounder between HH-EI trajectory and mental health, since we assume it to be highly associated with HH-EI trajectory and mental health as well [55, 56].

### Sequence analysis

Sequence analysis has a long tradition in social research [57]. It is a frequently used method for the purpose of capturing HH-EI trajectories over multiple years [58, 59]. The explanation for this is straightforward: the number of potential HH-EI trajectories is so great that they cannot be evaluated individually. Instead, sequences analysis compares the unique history of each individuals’ status (element variable) with all found records on the status in the given data. Then, different algorithms and clustering strategies are employed to assess the set of sequences that are most closely aligned with the individual in question, in comparison to the overall data set [60]. We used Stata (18.5) together with the SADI sequence analysis ado [61] and compared three different algorithms for matching, with the most routinely used optimal matching algorithm (OMA), Halpin's duration-adjusted OMAV [61], and the time warp edit distance (TWED) [62], which emphasizes transition and timing of switches in the element variable. Since Germany is considered a modified male-breadwinner labor market [17], sequences analyses were also performed gender stratified. We evaluate the optimal number of clusters using the Duda-Hart index since it has been found to be the most consistent measure when using the Ward's-Clustering method [63].

### Longitudinal analysis and periodical DiD-estimation

We applied rigorous panel data analysis and quasi-experimental DiD-estimation. The aims were to: (A) longitudinally estimate the social gradient in mental health between the found HH-EI trajectories; and: (B) to identify the periodical differences within the HH-EI trajectories before, during, and after the COVID-19 pandemic.

In the intervention model we estimated the probability of being observed in the intervention group (${P(T}_{i}=1$)) based on a vector of covariates (${V}_{i}$). The resulting inverse-probability weight, adjusts each individual (${IPW}_{i}$) for the probability of being observed in the intervention group [64].

Equation 1: Intervention model: Inverse probability weight for being observed in the intervention group

$$IPW_i=\frac1{P\left(T_i=1\left|V_i\right.\right)}$$

Then, we estimated the outcome model, which was a generalized linear population-averaged panel-model on mental health ($$g(E\left[{Y}_{it}\right]$$) weighted for the IPW of Eq. 1. The outcome model integrates a three-way $$({\beta }_{7}\left({{S}_{i}G}_{i}{T}_{t}\right))$$ interaction between HH-EI trajectories ($${S}_{i})$$, sample ($${G}_{i})$$ and time ($${T}_{t}$$) and is additionally adjusted for a vector of confounders ($${Z}_{i}{\mathbf{\top }}_{\gamma })$$**.**

Equation 2: Population-averaged panel-data model with Gaussian distribution, link function and three-way-interaction

$$g(E\left[Y_{it}\right]=\beta_0+\beta_1S_i+\beta_2G_i+\beta_3T_{it}+\beta_4\left(S_iG_i\right)+\beta_5\left(S_iT_{it}\right)+\beta_6\left(G_iT_{it}\right)+\beta_7\left(S_iG_iT_{it}\right)+Z_{it}{\mathrm T}_\gamma+\in_{it}$$

Finally, the DiD-estimates for a given HH-EI trajectory is simply the difference between the marginal effects at time ($${T}_{t}$$) between intervention and control group ($${G}_{i}=1|{G}_{i}=0,t)$$.

Equation 3 Difference-in-Difference estimate as the difference of the marginal effects

$$DiD_{ME}=\triangle\left[\frac{\partial g^{-1}\left(E\left[Y_{it}\right]\right)}{\partial S_{ik}}\right]G_i=1\left|G_i=0,t\right.$$

As an example, the DiD-estimate of the COVID-19 period is the difference of the marginal effects from the HH-EI trajectories at t3 between the control sample with the “fake” intervention period (per-COVID-19; C_t3_/wave 2016) and the intervention sample (per-COVID-19; T_t3_/wave 2020). The control sample mimicking the intervention sample, enables the evaluation of the pre-intervention differences in the outcome. In the event that such pre-intervention differences exist, the parallel trend assumption of the DiD approach is likely to be violated [44, 46].

To summarize, our empirical strategy involves five steps designed to compare HH-EI trajectories and their association with mental health before, during, and after the COVID-19 pandemic. First, we use GSOEP panel data from 2014 to 2022, dividing it into two samples: a placebo control sample (C) not exposed to COVID-19 (2014–2018) and an intervention sample (T) that includes the pandemic period (2018–2022). GSOEP’s rotating panel design minimizes overlap between the two groups, reducing bias from panel aging. This setup enables comparison of the associations before and after the onset of the pandemic. Second, we apply sequence analysis to identify five-year HH-EI trajectories. Third, to address potential selection bias—due to differences in participation or sample composition—we apply inverse probability weighting (IPW). Fourth, we estimate the association between HH-EI and mental health using a generalized panel model with interactions between HH-EI trajectory, time period (pre-, per-, post-COVID-19), and sample (C vs T). Finally, a difference-in-differences (DiD) approach assesses how the association between HH-EI and mental health changed across periods, using the pre-COVID-19 period as a baseline test. While we use quasi-experimental techniques to strengthen inference, our goal is to estimate relative changes in the association, and not to provide strong causal claims. These claims require the placement and satisfaction of further assumptions, that we discuss later on.

## Results

### Sequence analysis

The HH-EI trajectories have been cross-checked by applying and comparing the results of the three matching algorithms (OMA, OMAV, TWED). Appendix Table S1 details the fit statistics on the comparison. For OMA and OMAV we set the indelcost to amount twice the substation costs, and for TWED we set Lambda to 0.8 nu 0.1 to be more sensitive towards timing of the changes in the trajectories [61]. Overall, regardless of the algorithm, a five to seven cluster solution was considered optimal for males and females. However, the best performing algorithm was TWED for males and OMAV for females. Although a four-cluster to five-cluster solution would have been statistically as good as a six-cluster solution in most cases, we wanted to emphasize the nuances in the trajectory and provide some comparability across gender, and therefore decided to use the six-cluster solution. Additionally, Figure S1 further illustrates the differences in the sequences of the element variable for males and females in the intervention and placebo control samples.

**Fig. 2:**
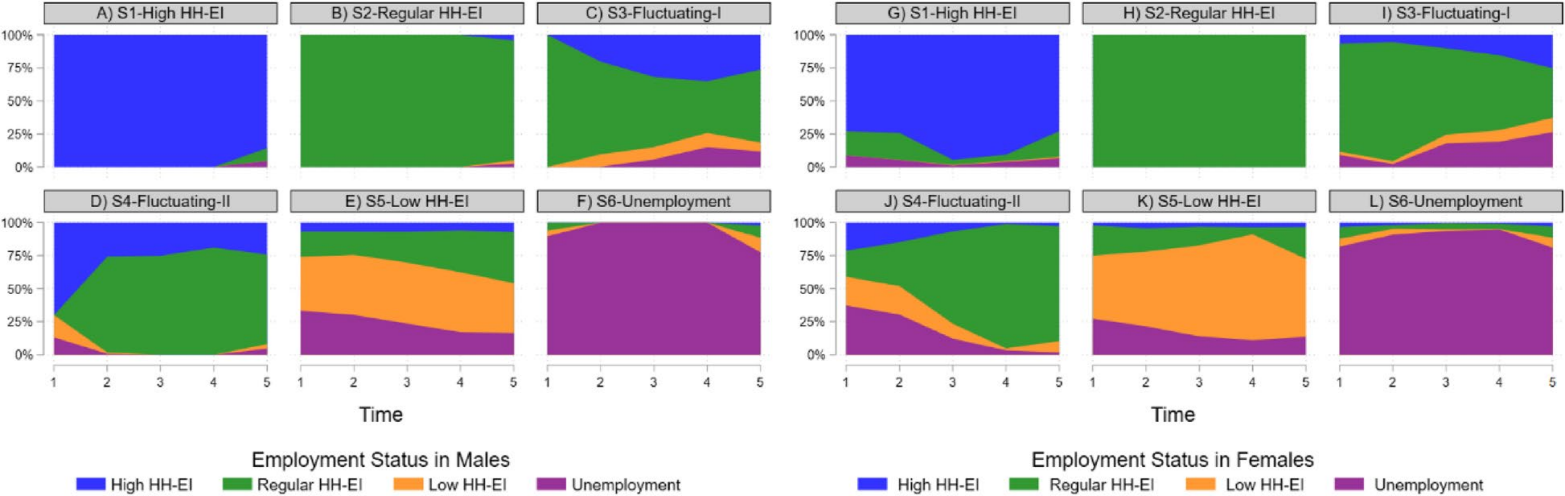
Proportional composition of element variable in the HH-EI trajectories S1-6. Chronograms show the distributional properties of the element variable within the HH-EI trajectories for males (**A**-**F**) and females (**G**-**L**)

Figure 2 illustrates the resulting cluster-solutions as chronogram. The chronograms represent the proportional composition of the element variable (HH-EI) over the five waves (t) in each HH-EI trajectory (cluster). Although both genders have comparable HH-EI trajectories, for males there is an all-around higher prevalence of high income and regular income in the fluctuating trajectories (S3-S4) and even in the mostly low-income group (S5) (see also, Figure S1.). When comparing trajectories S4 and S5 between males and females (Fig. 2D-E with Fig. 2J-K), gendered HH-EI trajectories become apparent.

### Descriptive statistics

Table 1 contains the descriptive statistics of the main variables and covariates between the control and intervention samples for males and females at baseline (t1). The element variable constituting the sequence variable varies between samples and gender. In the not exposed to COVID-19 sample (placebo control), for both genders, the share of high income was lower and the share of unemployed higher. The gender differences in unemployment and part-time employment (e.g., see also female unemployment 24.85%; part-time employment 32.25%; male unemployment 17.19%, part-time employment 5.10%, see also Table S2) confirm the modified male-breadwinner model of Germany. In addition, the average HH-EI when being unemployed was 1,303€ for females, while for unemployed males the average HH-EI was 1,181€ (see Table S3). The HH-EI trajectory also reflects this difference with males being less prominent in the low HH-EI trajectory (S5). Though, comparing the trajectories directly between gender is somewhat lacking because their composition is based on the gender specific trajectories (see also, Fig. 2 and Appendix Fig. 1S). There are no descriptive differences in mental health at baseline between the samples, but there are differences in gender with females reporting lower mental health than males. The mean age at the start of the trajectory varied between 41 to 43. Some differences between the exposed to COVID-19 (intervention) and not exposed to COVID-19 (placebo control) samples are observable towards having higher education in the intervention sample.

**Table 1:** Descriptive statistics for males and females in intervention and control sample

	Males		Females	
	Exposed to COVID-19 (Intervention) *N* = 3,724	Not exposed to COVID-19 (Placebo Control) *N* = 5,199	Exposed to COVID-19 (Intervention) *N* = 4,616	Not exposed to COVID-19 (Placebo Control) *N* = 6,670
Variables	Mean (SD)/%	Mean (SD)/%	Mean (SD)/%	Mean (SD)/%
Element Variable				
High-Inc	16.03%	14.00%	13.52%	11.08%
Reg.Inc	55.75%	61.51%	52.58%	52.01%
Low-Income	11.04%	10.02%	9.06%	10.49%
Unemployed	17.19%	14.46%	24.85%	26.42%
Sequence Variable (Predictor)				
S1-High HH-EI	6.44%	7.96%	10.75%	11.69%
S2-Regular HH-EI	40.74%	43.72%	32.84%	30.91%
S3-Fluctuating I HH-EI	22.80%	19.98%	25.78%	25.85%
S4-Fluctuating II HH-EI	9.10%	7.73%	11.27%	11.41%
S5-Low HH-EI	10.23%	7.62%	14.95%	15.47%
S6-Unemployment	10.69%	12.98%	4.42%	4.66%
Outcome				
Mental Health (MCS)	51.3 (9.51)	51.4 (9.0)	49.4 (10.2)	49 (9.92)
Covariates				
Physical Health (PCS)	52.4 (8.7)	52.2 (8.73)	50.8 (9.55)	51.3 (9.33)
Net-Equivalence-HH-Income	1985 (1,222)	1,812 (1,171)	1,919 (1,208)	1,695(1,016)
ISEI (Socioeconimc Status)	48.4 (21.4)	46.3 (21.5)	48.7 (19.3)	45.7 (19.6)
Age	43.1 (11.7)	42.2 (11.4)	42.3 (11.3)	41.4 (10.8)
Number of Children	0.73 (1.08)	0.78 (1.07)	0.714 (0.99)	0.814 (1.05)
CASMIN-Classification				
Low	26.10%	31.41%	20.69%	24.21%
Middle	41.00%	41.95%	50.37%	50.84%
High	32.89%	26.64%	28.94%	24.95%
German Citizenship	82.60%	90.25%	87.95%	88.98%
Married	59.85%	62.72%	55.91%	59.22%
Partnership	19.87%	17.85%	23.14%	19.78%
No Partnership	17.45%	16.00%	12.09%	12.11%
Divorced/Widowed	2.82%	3.42%	8.86%	8.89%
Change in Job	13.72%	11.18%	14.06%	13.67%

In Appendix Table S4, we evaluated the selectivity due to missing values between the theoretically available information on the variables at each episode (with valid entries for the respective variable) and the information lost due to having full information on the main variables [65]. We observe moderate selectivity towards older age with Cohen's D ~ 0.200 in the placebo control sample and low selectivity towards higher mental health in the intervention sample (Cohen's D = 0.100), which was lower in the control sample (Cohen's D = 0.050).

### Main results

Table 2 evaluates the performance of the intervention model by reporting the distributional properties of the IPW, as well the pseudo-R^2^ statistic. Table 2 also presents the results of the outcome model for both genders. The outcome model mainly consists of the three-way interaction between HH-EI trajectory—group—and time (Eq. 2). The single coefficients for the HH-EI trajectories represent the level difference in mental health of the respective HH-EI trajectories compared to the high-income trajectory (S1), when having the interaction variables set to the reference values (control sample and pre-COVID-19). For both genders, the single coefficients of the HH-EI trajectories indicate a significant gradient in mental health towards increased differences at low HH-EI or fluctuating HH-EI.

**Table 2:** Results of the Outcome and Intervention Model population-averaged

	Outcome Model	
Main Variables	Males	Females
	Coeff [C.I.]	Coeff [C.I.]
High HH-EI trajectories S1 = Reference		
S2-Regular HH-EI	−1.312^**^	−1.829^***^
	[−2.234,−0.390]	[−2.639,−1.020]
S3-Fluctuating I HH-EI	−1.457^**^	−2.593^***^
	[−2.512,−0.402]	[−3.519,−1.668]
S4-Fluctuating II HH-EI	−0.730	−1.797^***^
	[−1.973,0.514]	[−2.745,−0.849]
S5-Low HH-EI	−2.455^***^	−3.374^***^
	[−3.531,−1.379]	[−4.469,−2.279]
S6-Unemployment	−4.729^***^	−3.725^***^
	[−6.278,−3.179]	[−4.725,−2.725]
Intervention Group (Control = Reference)	0.636	0.158
	[−0.948,2.219]	[−0.950,1.267]
per-COVID-19 (pre-COVID-19 = Reference)	0.456	0.002
	[−0.351,1.264]	[−0.717,0.721]
post-COVID-19 (pre-COVID-19 = Reference)	0.169	−0.514
	[−0.698,1.036]	[−1.227,0.200]
Three-way interaction		
S2#Intervention#per-COVID-19	−0.940	0.245
	[−2.546,0.665]	[−1.084,1.575]
S2#Intervention#post-COVID-19	−0.056	−0.710
	[−1.856,1.744]	[−2.229,0.809]
S3#Intervention#per-COVID-19	−0.598	0.478
	[−2.498,1.301]	[−1.101,2.056]
S3#Intervention#post-COVID-19	0.590	−0.579
	[−1.519,2.700]	[−2.294,1.137]
S4#Intervention#per-COVID-19	−0.869	−0.242
	[−2.822,1.084]	[−1.760,1.276]
S4#Intervention#post-COVID-19	0.583	−0.215
	[−1.586,2.752]	[−1.919,1.488]
S5#Intervention#per-COVID-19	−0.903	0.198
	[−2.716,0.910]	[−1.530,1.927]
S5#Intervention#post-COVID-19	0.044	−0.959
	[−1.957,2.044]	[−2.913,0.995]
S6#Intervention#per-COVID-19	1.599	1.525
	[−0.716,3.913]	[−0.040,3.091]
S6#Intervention#post-COVID-19	0.902	0.962
	[−1.634,3.439]	[−0.735,2.658]
Model Fits:		
Wald Chi^2^ (54)	697.20	1010.49
Prob > chi2	= 0.000	0.000
Number of Observations	23,679	29,979
Number of Groups	8,932	11,286
Scale Parameter	83.41	99.78
	Intervention Model	
IPW (Mean/SD)	0.998 (0.437)	1.000 (0.337)
Pseudo-R^2^	0.126	0.097

Outcome Model: population-averaged panel-data models with gaussian distribution and link function, Huber/White sandwich variance estimator, Quasi-Likelihood Estimation and IPW applied. Intervention: Probability Model on Intervention vs. Control group

^*^p < 0.05, **p < 0.01, ***p < 0.001

For females this gradient is more pronounced as shown by the higher and significant coefficients compared to males (e.g., for males in S5 = −2.455^****^; for females in S5 = −3.374^****^). The model-fit statistics imply a higher explanatory power for the male outcome model (scale parameter = 83.41) than for the female outcome model (scale parameter = 99.78). Similarly, the intervention model, although having produced stable IPWs, shows a higher pseudo-R^2^ for males than for females (0.126 vs 0.097). Although the three-way interaction parameters in Table 2 are insignificant. Though, the three-way interaction parameters are necessary to obtain the marginal effects and their differences in the HH-EI trajectories they are not of primary interest. Table S5 in the Appendix contains further information on the covariate coefficients included in the outcome model.

**Fig. 3:**
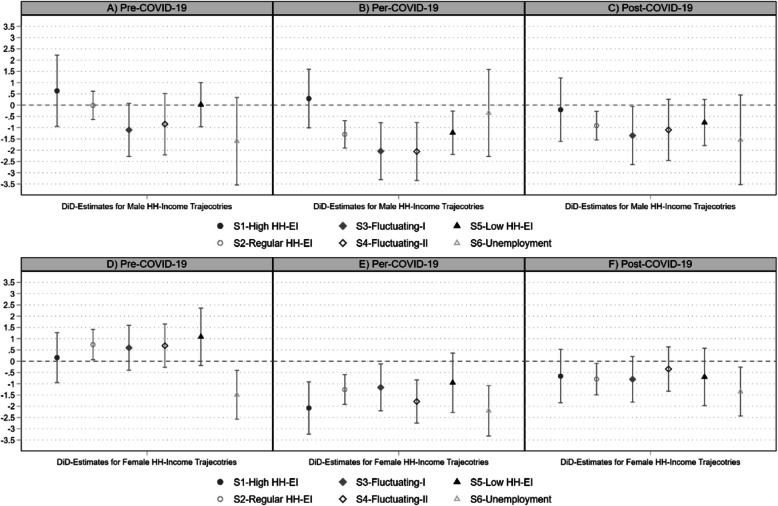
DiD-estimates of HH-EI trajectories at pre-, per-, and post-COVID-19 period between intervention and control sample. DiD-estimates of Eq. 3 for males (**A**-**C**) and females (**D**-**F**)

The main research aim is to investigate the relative differences in mental health for the HH-EI trajectories in the pre-, per-, and post-COVID-19 context. For this purpose, Fig. 3 and Table 3 present the DiD-estimates of the marginal effects (Eq. 3) derived from the coefficients of the HH-EI trajectories (S1-S6) in Table 2 (Eq. 2). The three-way interaction terms in Table 2 are not statistically significant, which indicates that we do not observe consistent conditional differences in mental health. However, the non-significance of interaction coefficients does not imply that conditional effects are absent altogether [66]. Importantly, statistically significant differences may still emerge at specific combinations of HH-EI trajectories, intervention status, and time points. Therefore, we rely on marginal effects to explore these conditional patterns in more detail.

**Table 3:** Difference-in-Difference-Estimates between Intervention and Control Sample for Males and Females

	Males Intervention vs. Control			Females Intervention vs Control		
DiD	Pre-Covid	COVID	Post-Covid	Pre-Covid	COVID	Post-Covid
S1	0.636	0.292	−0.203	0.158	−2.079^***^	−0.663
	[−0.948,2.219]	[−1.007,1.592]	[−1.610,1.204]	[−0.950,1.267]	[−3.240,−0.918]	[−1.850,0.524]
S2	−0.013	−1.297^***^	−0.908^**^	0.736^*^	−1.256^***^	−0.795^*^
	[−0.638,0.612]	[−1.906,−0.688]	[−1.545,−0.271]	[0.064,1.409]	[−1.916,−0.597]	[−1.497,−0.093]
S3	−1.101	−2.043^**^	−1.350^*^	0.595	−1.165^*^	−0.805
	[−2.279,0.077]	[−3.307,−0.779]	[−2.637,−0.063]	[−0.401,1.590]	[−2.206,−0.125]	[−1.817,0.206]
S4	−0.844	−2.057^**^	−1.101	0.688	−1.791^***^	−0.349
	[−2.209,0.520]	[−3.344,−0.771]	[−2.456,0.255]	[−0.271,1.647]	[−2.748,−0.835]	[−1.333,0.635]
S5	0.022	−1.224^*^	−0.773	1.081	−0.958	−0.699
	[−0.959,1.004]	[−2.188,−0.260]	[−1.794,0.247]	[−0.192,2.354]	[−2.278,0.361]	[−1.974,0.575]
S6	−1.602	−0.347	−1.538	−1.491^**^	−2.204^***^	−1.351^*^
	[−3.544,0.341]	[−2.277,1.584]	[−3.528,0.451]	[−2.577,−0.406]	[−3.318,−1.090]	[−2.437,−0.266]

Confidence Intervals in Brackets below DiD-estimates

^*^p < 0.05,** *p* < 0.01,*** *p* < 0.001

The DiD-estimates are calculated at each period (pre-/per-/post-COVID-19) for each HH-EI trajectory as the difference in the marginal effects between the intervention and control sample (Eq. 3). Therefore, in Fig. 3, a deviation below the dashed line (zero) indicates a decline in mental health for the intervention group during the respective period. As a result, in the pre-COVID-19 period, there should be no significant differences, as these would imply unaccounted for pre-intervention differences between the samples.

For males in Fig. 3 (A-C), the pre-intervention DiD-estimates are insignificant (see Fig. 3A and Table 3). However, during the COVID-19 pandemic (Fig. 3B), mental health decreases significantly for HH-EI trajectories S2-S5 by up to ¼ SD-unit in MCS (e.g., for DiD_S3per_ = −2.043^**^). Only the poles, mostly high HH-EI (S1) and mostly unemployed (S6), display no decrease in males’ mental health during the COVID-19 pandemic. In the post-pandemic period (Fig. 3C), these decreases in mental health partially diminish. Still, for the regular HH-EI trajectory (S2) and the fluctuating I HH-EI (S3) the deviation below zero remain significant (e.g., DiD_S3post_ = −1.350^*^). Additionally, we also provide non-gender stratified visualization of the DiD-estimates in Appendix Figure S2, indicating a significant negative DiD-estimates across all trajectories during the COVID-19 pandemic, and generalized slight recovery tendency post-pandemic, with remaining negative DiD-estimates for trajectories S2, S3 and S6.

For females, there are some significant deviations from zero in the pre-COVID-19 period (see Fig. 3C, S2 and S6). Specifically, for the mostly regular HH-EI trajectory (S2), the baseline levels of mental health are higher in the intervention sample, and for the unemployed trajectory (S6), these levels are already lower. Despite this, during the COVID-19 pandemic, mental health declines significantly for all HH-EI trajectories except for S5. In contrast to males, this decrease is also present for females in the high HH-EI trajectory (DiD_S1per_ = −2.079^***^) and for the unemployed trajectory (DiD_S6per_ = −2.204^***^). Yet, similarly to males, the post-pandemic DiD-estimates are mostly weaker and insignificant, except for females in the regular HH-EI trajectory (DiD_S2post_ = −0.795^*^).

## Discussion

This study investigated the association between HH-EI trajectories and mental health in a pre-, per-, and post-COVID-19 context. Our findings confirmed a pronounced social gradient in mental health, with high HH-EI trajectories showing improved mental health and lower HH-EI or fluctuating trajectories having lower mental health. Depending on the HH-EI trajectory, there was a notable decline in mental health during the COVID-19 pandemic, with a reduction of approximately one-quarter of a standard deviation in mental health. We also found a partial, but not complete, return of the HH-EI trajectory mental health association two to three years after the onset of the pandemic. Our results also highlight stark gender differences, with females experiencing a steeper mental health gradient, a higher prevalence of unemployment and low HH-EI trajectories, and a more general decline in mental health across most HH-EI trajectories during the pandemic.

The results of this study are presented against a robust methodological framework that aims at approximating a causal estimation, consisting of five steps. (I) Quasi-experimental assignment of the GSOEP respondents into an exposed to (T) and not exposed to (C) COVID-19 pandemic sample; (II) sequence analysis to obtain the HH-EI trajectories; (III) reduction of non-random differences between T and C sample through IPW calculation; (IV) an IP-weighted panel model consisting of a three-way-interaction between HH-EI trajectory – sample – and period. Finally, (V) a DiD estimation that quantifies the relative changes in the HH-EI trajectory – mental health association for each period.

These steps were performed stratified by gender, and our results confirm the necessity of gender-stratification. Especially in Germany, with a persisting modified male-breadwinner model [17], the comparison between Fig. 2B and Fig. 2E reveals that, even within the high HH-EI trajectory (S1), females experienced a significant mental health decline, as indicated by the DiD estimates of −2.079^***^, whereas males in the similar trajectory (S1) remained stable. This disparity may be explained by gendered burdens, with predominantly females having caregiving and domestic responsibilities during the pandemic, as highlighted by recent studies [54, 67, 68]. Such persistent gendered caregiving roles may explain the notable decline in mental health among females participating in the labor market and having a high HH-EI, as these responsibilities appear to be unaffected by labor market participation. An advantage of using HH-EI instead of individual income is, that individual income measures would likely lead to misclassifying economically dependent spouses or partners as living in poverty, despite their access to substantial shared household income [47], thus, obscuring these gendered differences.

For males, however, declines in mental health were also evident during the pandemic, but those within the high HH-EI (S1) and unemployed (S6) trajectories showed resiliency towards mental health losses. The finding that males at opposite ends of the employment spectrum, high HH-EI (S1) and unemployment (S6), exhibit resilience to mental health decline during the COVID-19 pandemic may appear counterintuitive. Though, there are plausible theoretical and empirical arguments in favor of its plausibility. For individuals in the high HH-EI trajectory (S1), greater financial security and access to robust buffering resources mitigate the mental health impact of the crises, as these resources have been found to be highly gendered in Germany [69]. Conversely, arguing with the conservation of resources theory [4], individuals in the unemployment trajectory (S6) may have adapted to fewer resources, could experience less disruption during crises, compared to those who have more resources to lose during acute disruption, resulting in reduced sensitivity to the COVID-19 pandemic. In addition, the social norm of unemployment hypothesis [70, 71] provides a complementary explanation: when unemployment becomes the norm, being unemployed may represent less of a deviation from societal expectations. As a result, the psychological burden associated with unemployment, such as feelings of shame or social exclusion, may be reduced, thereby attenuating the negative mental health consequences. In this context, there is also evidence that unemployment is negatively associated with health outcomes only in cross-section, but not longitudinally [72]. Figure 2F supports this explanation; the consistently unemployment trajectory (S6), shows no significant deviation in mental health for the pre-, per-, post-pandemic period.

The more generalized impact of crises such as the COVID-19 pandemic on females found in the current study is supported by explanations from previous research [27]. Overall, the results suggest greater heterogeneity in the relationship between HH-EI trajectory and mental health among females, mediated or moderated by additional factors. These include gendered caregiving roles and domestic responsibilities [25, 73], which may further amplify disparities in mental health outcomes during crises like the COVID-19 pandemic. To enable more precise policy interventions, future research should expand on the employment-gender-mental health intersections with a focus on time-varying mediation and moderation processes, such as given by economic worries, health behaviors work-related stressors and care-burdens [31, 55, 22].

Germany's status as a social welfare state provides baseline security through unemployment benefits and social assistance, which help mitigate external shocks [74, 75]. Another critical factor is the presence or absence of workplace stressors. The COVID-19 pandemic notably amplified such stressors, particularly for close-contact and precarious occupations, by increasing the risks of infection and job loss due to measures like shutdowns and lockdowns and having to rapidly adapt to new technologies and protocols at work [76]. These factors may be reflected in the observed decline in mental health among individuals within the fluctuating trajectories (S2-S3), as their exposure to labor market disruptions during the pandemic heightened threats of downward mobility. Similarly, for individuals already in a lower HH-EI trajectories (S4-S5), the pandemic likely increased their vulnerability, reinforcing the so-called “precarity trap” [77]. Concerning this precarity trap in more depths, investigations of the employment quality aspects and forms of precarious employment show an immediate effect on health [19, 78, 79]. Further research within this framework is needed to investigate the accumulating disadvantages and the periodical variation within crises, such as the COVID-19 pandemic and economic crises.

Concerning the methodological framework in general, for a causal interpretation the outlined quasi-experimental design requires several assumptions to hold, most importantly exogeneity of the intervention assignment (no systematic selection), parallel trend assumption [45], homogeneity of the intervention effects; and no post-intervention confounding. Concerning the exogeneity of the treatment assignment, the IPW successfully reduced selectivity between T and C sample, with only low selectivity towards older and higher mental health, arguing for a conservative bias (see also: Appendix Table S4). Unobserved confounding might still exist in forms of individual-level psychological factors affecting mental health and HH-EI, as well as having access to further wealth assets that are not part of the household income. Concerning the assumption of no post-intervention confounding, the intervention sample may still suffer from bias, since the pandemic was marked by heterogeneous infections, and pandemic-response measures. Although, we used the information of the interview month as covariate to capture these dynamics; heterogeneity is inherent. Furthermore, regarding the post-pandemic period, similar post-intervention confounding is present by the Russian invasion of Ukraine in February 2022, which as has led to subsequent economic insecurities in the following months, especially in Germany. Having quarterly waves instead of annual and longer post-intervention periods would allow further identification and modelling of the heterogeneity due to time-variability [80]. Regarding the parallel trend assumptions, we provide a rigorous test of the parallel trend assumption through inspection of pre-intervention differences with a placebo-control design [46, 80], and our results suggest only few pre-intervention differences exist (see Fig. 3A). A further comparison of the unweighted and uncontrolled model with the weighted and controlled models (comparison of Table S6 with Table 3) indicates strong reductions in pre-treatment differences and avoidance of underestimation of the DiD-estimation.

The intervention model estimated the likelihood of selection into intervention or control groups and mitigated the selectivity, as evident by the increased pre-intervention differences and an underestimation of the DiD-estimates (see Appendix Table S5). The panel outcome model consisted of a population-averaged model with a three-way interaction among HH-EI trajectory, group, and time. Although the interaction parameters themselves were statistically insignificant, the derived marginal effects provided crucial insights. Specifically, the DiD-estimates (Eq. 3) highlighted differences within the effect of the HH-EI trajectories on mental health during and even after the COVID-19 pandemic. A simple two-way fixed effects regression instead of the DiD-estimation might appear more convenient in many cases, in our case; however, it was not computable because, even though the HH-EI trajectories consist of time-varying information on employment status, the trajectories themselves are time constant.

Furthermore, the sequence analysis highlighted a clear segmentation of HH-EI trajectories and revealed a strong mental health gradient across these trajectories, independent of the analyzed periods. We encourage future research to extend these findings by examining the timing of transitions and their effects in the post-pandemic period. For instance, how does the mental health impact of unemployment vary when it initially occurs during the COVID-19 pandemic? Investigating such specific transitions could also optimize the timing and targeting of support interventions (e.g., financial reliefs) for populations within certain household levels during times of crisis.

The findings of this study carry important implications for public health and labor market policies in Germany and comparable welfare states. First, the mental health burden during the COVID-19 pandemic was most pronounced among individuals in regular and fluctuating income trajectories, groups that are typically unconsidered by welfare policies. As such, social protection policies should be recalibrated to include individuals in these income trajectories, particularly in times of economic or public health crises. The pronounced and persistent gender disparities in mental health trajectories further call for an intersectional policy approach that acknowledges the cumulative disadvantage of women in both labor market participation and care responsibilities. To address this, policy efforts must focus on expanding affordable and accessible childcare, strengthening paid family leave, and promoting equitable intra-household division of care labor. Such measures would not only reduce role strain but also enhance women's resilience to labor market shocks. Lastly, the study underscores the importance of mental health as an integrated dimension of crisis preparedness and response. While Germany’s social welfare system provides a degree of financial stability, it does not fully buffer against psychosocial stressors in the face of systemic shocks. These shocks can lead to diminishing mental health and growing social inequalities, ultimately undermining progress toward achieving SDG 3 and SDG 10.

## Conclusions

This study draws attention to the considerable challenges Germany is facing in achieving Sustainable Development Goals 3 (Good Health and Well-Being) and 10 (Reduced Inequalities) before, during and after the COVID-19 pandemic. The findings reveal that health inequalities, which increased in the COVID-19 pandemic, only partially recovered in the post-pandemic period. There is a strong gradient in mental health by the household income level, with gendered vulnerability among females, particularly in times of crisis, underlining persistent inequalities. While Germany's social security system seems to provide some buffering against these impacts, new vulnerable strata emerged, especially among individuals within regular household income levels and experienced household income losses, indicating limitations in the existing safety nets. The found stark gender differences, also call for a reevaluation of pandemic response plans to better address the gendered dynamics of health and employment [81].

## Supplementary Information

Supplementary Material 1

## Data Availability

The German Socio-Economic Panel (GSOEP) is available for scientific purposes for academic users within and outside of Germany. Data access can be requested by the German Institute of Economic Research (Deutsches Institut für Wirtschaftsforschung- DIW) at: https://www.diw.de/en/diw_01.c.357906.en/soep_order_form.html. For general information, see also: http://www.diw.de/soep.

## Abbreviations

HH-EI: Net Household Equivalized Income
SDG: Sustainable Development Goals
SDG3: Sustainable Development Goals health and wellbeing
SDG10: Sustainable Development Goals less social inequality
IPW: Inverse-Probability-Weighting
DiD: Difference-in-Difference
GSOEP: German Socioeconomic Panel
(T): Intervention Sample; Respondents exposed to COVID-19 pandemic
(C): Placebo Control Sample; Respondents not exposed to COVID-19 pandemic
MCS: Mental Component Summary
SF-12v2: Short Form-12 Health Survey
SD: Standard Deviation
PCS: Physical Component Score
S1-6: HH-EI trajectories 1 to 6
OMA: Optimal Matching Algorithm
OMAV: Halpin's duration-adjusted Optimal Matching Algorithm
TWED: Time-Warp Edit Distance Algorithm
