# Supplementary material for: Household-income trajectories and mental health inequalities in Germany before, during, and after the COVID-19 pandemic: a quasi-experimental panel study
Source: Int J Equity Health. 2025 May 19;24:143. doi: 10.1186/s12939-025-02507-1 (PMC12090502; doi:10.1186/s12939-025-02507-1)
Supplement: Supplementary file 1 — Supplementary Material 1 [file 12939_2025_2507_MOESM1_ESM.docx]

# **Online Appendix**

Table S1 Performance evaluation of sequence analysis with OMA, OMAV and TWED algorithm

| Males | OMA  Indelcost (1) subcost (0.5) | | | OMAV  Indelcost (1) subcost (0.5) | | | TWED  Lambda (0.8) nu (0.1) | | |
| --- | --- | --- | --- | --- | --- | --- | --- | --- | --- |
| No. Cluster | Duda-hart Je(2)/Je(1) | pseudo T² | pseudo-F | Duda-hart Je(2)/Je(1) | pseudo T² | pseudo-F | Duda-hart Je(2)/Je(1) | pseudo T² | pseudo-F |
| 1 | 0.929 | 686.710 |  | 0.967 | 271.470 |  | 0.905 | 838.830 |  |
| 2 | 0.308 | 7482.150 | 686.520 | 0.935 | 630.650 |  | 0.873 | 1317.970 |  |
| 3 | 0.707 | 801.160 | 2924.870 | 0.332 | 6728.120 | 630.560 | 0.234 | 10925.190 | 1318.480 |
| 4 | 0.868 | 870.520 | 2053.880 | 0.795 | 507.680 | 2686.850 | 0.804 | 652.360 | 3098.620 |
| 5 | 0.974 | 60.030 | 1977.770 | 0.891 | 693.580 | 1855.870 | 0.992 | 47.870 | 2211.840 |
| ^*^6 | 0.757 | 445.750 | 1612.740 | 0.955 | 105.300 | 1723.610 | 0.982 | 35.080 | 1687.690 |
| 7 | 0.972 | 30.180 | 1482.300 | 1.000 | 0.200 | 1430.400 | 0.956 | 87.770 | 1368.330 |
| 8 | 0.882 | 137.380 | 1282.040 | 0.567 | 996.910 | 1191.950 | 0.654 | 414.060 | 1153.260 |
| 9 | 0.996 | 4.590 | 1133.490 | 0.723 | 527.060 | 1056.600 | 0.950 | 57.130 | 1094.140 |
| 10 | 0.719 | 322.810 | 1008.940 | 0.581 | 367.350 | 1047.830 | 0.968 | 36.470 | 977.430 |
| Females | OMA | | | OMAV | | | TWED | | |
| No. Cluster | Duda-hart Je(2)/Je(1) | pseudo T² | pseudo-F | Duda-hart Je(2)/Je(1) | pseudo T² | pseudo-F | Duda-hart Je(2)/Je(1) | pseudo T² | pseudo-F |
| 1 | 0.817 | 2546.890 |  | 0.854 | 1940.860 |  | 0.781 | 3194.630 |  |
| 2 | 0.413 | 7050.450 | 2547.180 | 0.677 | 2999.420 | 1941.300 | 0.745 | 2008.210 | 3194.710 |
| 3 | 0.841 | 677.840 | 4427.880 | 0.499 | 2297.310 | 2054.140 | 0.492 | 4314.460 | 2974.290 |
| 4 | 0.838 | 1241.260 | 3118.020 | 0.820 | 1532.470 | 2198.050 | 0.949 | 131.520 | 4047.080 |
| 5 | 0.963 | 69.440 | 2999.290 | 0.918 | 465.440 | 2365.880 | 0.693 | 2483.760 | 3081.200 |
| ^**^6 | 0.907 | 299.660 | 2414.750 | 0.985 | 27.600 | 2676.450 | 0.812 | 393.350 | 3562.090 |
| 7 | 0.787 | 350.590 | 2180.620 | 0.996 | 8.770 | 1740.610 | 0.814 | 305.590 | 3195.160 |
| 8 | 0.806 | 364.330 | 1911.690 | 0.875 | 67.840 | 1495.250 | 0.858 | 782.900 | 2184.350 |
| 9 | 0.992 | 11.790 | 1826.000 | 0.587 | 761.530 | 1328.220 | 0.868 | 957.960 | 2720.170 |
| 10 | 0.992 | 11.900 | 1628.230 | 0.793 | 300.990 | 1111.490 | 0.934 | 55.350 | 2469.610 |

Note. ^*^Male 6-Cluster solution and TWED algorithm used. ^**^Female 6-Cluster solution with OMAV algorithm used. The Duda-Hart Index (Je(2)Je(1)) comparison of within-cluster variation. Higher values indicate adding more clusters to not improve. $\mathrm{pseudo}T^{2}=\frac{sum of squared distances between clusters}{sum of squared distances within clusters}$, lower argue for remain in cluster solution. Pseudo-F measure for distinction of clustering, higher values indicate better distinction.

Figure S1 Sequence plot of employment status for gender and sample


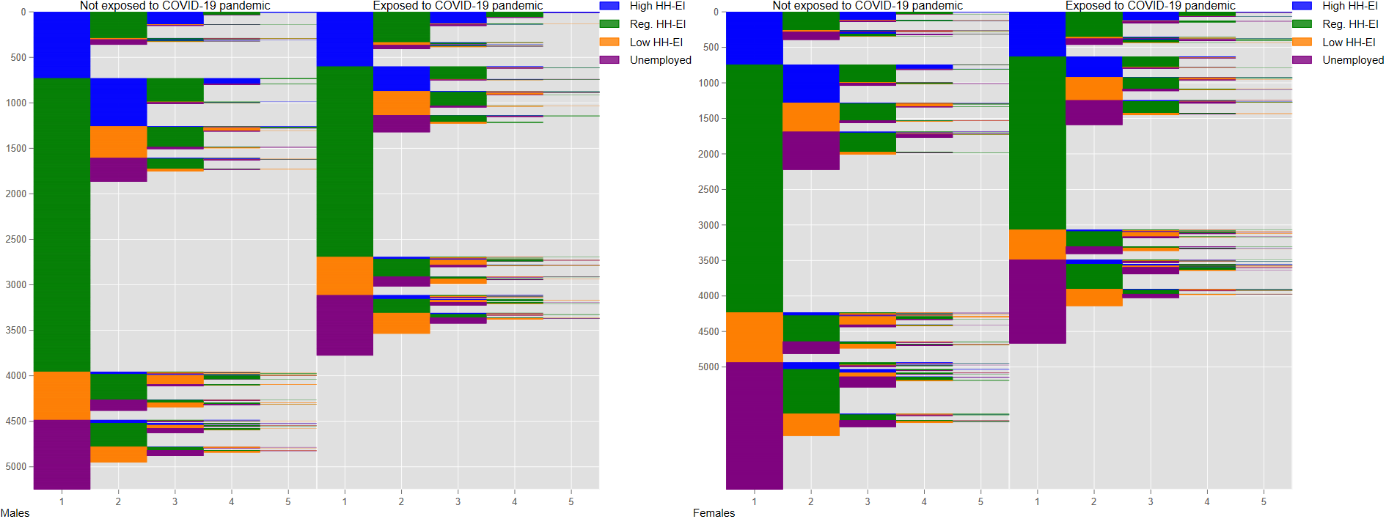


**Table S2 Distribution of Employment statuses and parental leave between genders**

| Employment Status at baseline | Males | *Parental Leave | Females | *Parental Leave |
| --- | --- | --- | --- | --- |
| [1] Fully employed | 72.78% |  | 30.75 | 0.02% |
| [2] Part-time employment | 5.10% | 1.75% | 32.25 | 1.04% |
| [3] Training/apprenticeship | 2.52% |  | 1.97 |  |
| [4] marginally employed | 3.45 |  | 8.91 | 3.28% |
| [5] Partial retirement | 0.09 |  | 0.09 |  |
| [6] Voluntary military service | 0.01 |  | 0.00 |  |
| [7] FSJ/FOeJ/BFD | 0.21 |  | 0.14 |  |
| [8] Workshop for the disabled | 0.17 |  | 0.10 |  |
| [9] Unemployed | 15.60 | 1.29% | 25.78 | 17.22% |
| [10] Company internship | 0.07 |  | 0.01 |  |
| Total | 8,923  100.00 | 30  0.33% | 11,286  100.00% | 501  4.44% |

*Percentage within the respective employment status e.g., 1.75% of males in part-time employment were in parental leave, and 17.22% of all females in unemployment were in parental leave. In total only 0.33% of all males in parental leave and 4.44% of all females.

**Table S3 Mean HH-EI by income group and gender at baseline**

| Net-Household-Eq-Income  by group at baseline | Males | | Females | |
| --- | --- | --- | --- | --- |
|  | Mean | SD | Mean | SD |
| High | 3944.8 | 1925.1 | 3898.0 | 1692.7 |
| Regular | 1828.2 | 479.0 | 1806.7 | 476.3 |
| Low-Income | 846.5 | 184.4 | 856.4 | 174.9 |
| Unemployed | 1181.2 | 834.0 | 1303.2 | 929.1 |

**Table S4 Selectivity between full available information and analysis sample**

|  | Full available information | | | Analysis samples treatment sample | | |  |
| --- | --- | --- | --- | --- | --- | --- | --- |
| Pre (t1) | Mean | SD | N | Mean | SD | N | Selectivity |
| MCS | 50.25 | 10.03 | 24,551 | 50.3 | 9.95 | 8340 | 0.010 |
| Age | 41.50 | 11.90 | 24,551 | 42.2 | 11.5 | 8340 | 0.060 |
| Per (t3) | Mean | SD | N | Mean | SD | N | Selectivity |
| MCS | 49.79 | 10.00 | 24,890 | 49.7 | 10 | 8340 | -0.010 |
| Age | 43.50 | 11.90 | 24,890 | 44.2 | 11.5 | 8340 | 0.060 |
| Post (t5) | Mean | SD | N | Mean | SD | N | Selectivity |
| MCS | 48.73 | 10.7 | 23,926 | 49.8 | 10.4 | 8340 | 0.100 |
| Age | 45.50 | 11.90 | 23,926 | 46.2 | 11.5 | 8340 | 0.060 |
|  | Full available information | | | Analysis samples Control sample | | |  |
| Pre (t1) | Mean | SD | N | Mean | SD | N | Selectivity |
| MCS | 50.09 | 9.75 | 23857 | 50.1 | 9.61 | 11869 | 0.001 |
| Age | 39.1 | 11.9 | 23857 | 41.7 | 11.1 | 11869 | 0.218 |
| Per (t3) | Mean | SD | N | Mean | SD | N | Selectivity |
| MCS | 50.53 | 10.19 | 23,344 | 51 | 9.68 | 11869 | 0.046 |
| Age | 41.1 | 11.9 | 23,344 | 43.7 | 11.1 | 11869 | 0.218 |
| Post (t5) | Mean | SD | N | Mean | SD | N | Selectivity |
| MCS | 50.25 | 10.03 | 24,551 | 50.3 | 9.75 | 11869 | 0.005 |
| Age | 43.1 | 11.9 | 24,551 | 45.7 | 11.1 | 11869 | 0.218 |

Note. Full available information consisting of all participant with valid entries at t. Analysis sample consisting individuals eligible for final analysis (panel participation, no missing values on main variables).

**Table S5 Covariate Coefficients of Table 2**

|  | Males | Females |
| --- | --- | --- |
| Age_spline1 | -0.330^***^ | -0.153^***^ |
|  | [-0.406,-0.254] | [-0.225,-0.080] |
| Age_spline2 | 0.803^***^ | 0.317^**^ |
|  | [0.564,1.042] | [0.089,0.544] |
| Age_spline3 | -3.322^***^ | -0.978 |
|  | [-4.683,-1.960] | [-2.265,0.309] |
| Age_spline4 | 4.723^***^ | 1.367 |
|  | [2.017,7.428] | [-1.211,3.944] |
| West-Germany (ref) | 0.000 | 0.000 |
|  | [0.000,0.000] | [0.000,0.000] |
| East-Germany | -0.444^*^ | 0.293 |
|  | [-0.857,-0.030] | [-0.097,0.682] |
| Partner not fully employed (ref) | 0.000 | 0.000 |
|  | [0.000,0.000] | [0.000,0.000] |
| Partner fully employed | 0.278 | 0.524^**^ |
|  | [-0.061,0.617] | [0.211,0.837] |
| 0.No migration background (ref) | 0.000 | 0.000 |
|  | [0.000,0.000] | [0.000,0.000] |
| 1.Migration background | 1.304^***^ | 1.471^***^ |
|  | [0.781,1.828] | [0.958,1.984] |
| 0.casmin (ref) | 0.000 | 0.000 |
|  | [0.000,0.000] | [0.000,0.000] |
| 1.casmin | 0.275 | 0.871^***^ |
|  | [-0.178,0.728] | [0.400,1.342] |
| 3.casmin | 1.090^***^ | 1.267^***^ |
|  | [0.551,1.630] | [0.705,1.830] |
| Married (ref) | 0.000 | 0.000 |
|  | [0.000,0.000] | [0.000,0.000] |
| In relationship (unmarried) | 0.170 | -0.980^***^ |
|  | [-0.275,0.616] | [-1.390,-0.571] |
| Single | -0.613^*^ | -1.424^***^ |
|  | [-1.200,-0.026] | [-1.981,-0.866] |
| Divorced/Widowed | -1.880^***^ | -1.785^***^ |
|  | [-2.815,-0.946] | [-2.400,-1.170] |
| No. of children in HH | 0.130 | 0.411^***^ |
|  | [-0.044,0.305] | [0.224,0.598] |
| isei08 | -0.011^**^ | 0.000 |
|  | [-0.020,-0.003] | [-0.009,0.009] |
| 1.Not employed | 0.000 | 0.000 |
|  | [0.000,0.000] | [0.000,0.000] |
| 2.Employed without change | 1.704^***^ | 1.043^***^ |
|  | [1.153,2.254] | [0.630,1.456] |
| 3.Employed without information on change | 1.961^**^ | 0.901 |
|  | [0.620,3.303] | [-0.394,2.195] |
| 4.Employed with change | 1.791^***^ |  |
|  | [1.212,2.369] | 1.355^***^ |
| 5.First-time employed | 1.924^*^ | [0.910,1.799] |
|  | [0.349,3.500] | 2.391^***^ |
| Physical health (PCS) | -0.179^***^ | [1.150,3.631] |
|  | [-0.197,-0.160] | -0.171^***^ |
| Constant | 70.719^***^ | [-0.187,-0.155] |
|  | [67.982,73.456] | 62.391^***^ |

* Note: * p<0.05, ** p<0.01, *** p<0.001; Confidence Intervals in Brackets below DiD-estimates.

**Figure S2: Non-gender stratified DiD-Estimates**


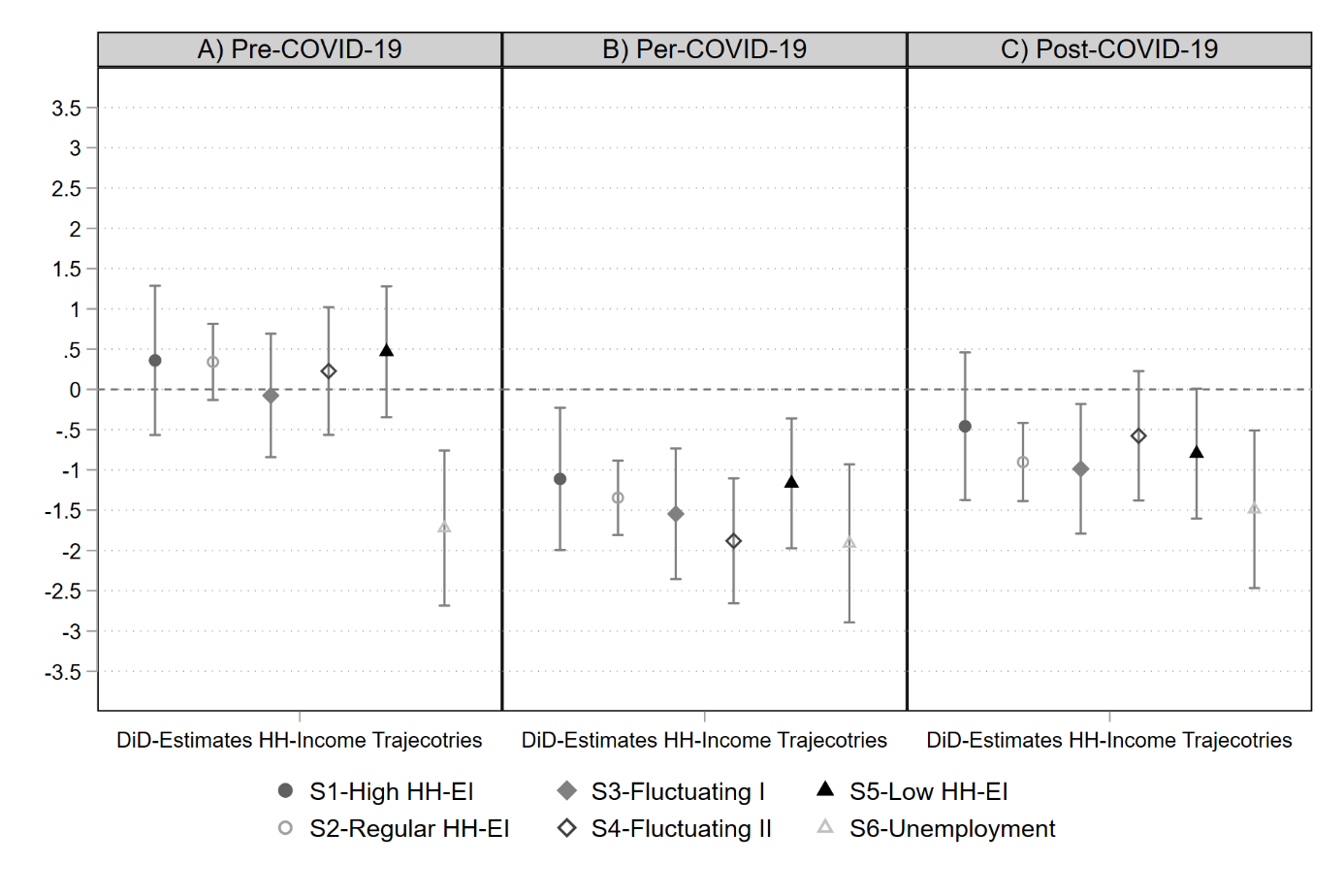


**Table S6 DiD-Estimates without weighting and control**

| Outcome Model | | | | | | |
| --- | --- | --- | --- | --- | --- | --- |
|  | Males | Treatment vs. Control | | Females | Treatment vs Control | |
| DiD | Pre-Covid | COVID | Post-Covid | Pre-Covid | COVID | Post-Covid |
| S1 | 1.028 | 0.173 | 0.066 | 0.175 | -1.981^***^ | -0.803 |
|  | [-0.200,2.256] | [-1.060,1.406] | [-1.224,1.356] | [-0.881,1.231] | [-3.089,-0.873] | [-1.960,0.354] |
| S2 | 0.204 | -1.078^***^ | -0.725^*^ | 0.635^*^ | -1.296^***^ | -0.774^*^ |
|  | [-0.340,0.749] | [-1.636,-0.519] | [-1.310,-0.140] | [0.002,1.268] | [-1.919,-0.674] | [-1.432,-0.117] |
| S3 | -0.596 | -1.440^*^ | -0.552 | 0.989^*^ | -1.008^*^ | -0.913 |
|  | [-1.711,0.519] | [-2.615,-0.264] | [-1.732,0.628] | [0.006,1.972] | [-1.995,-0.020] | [-1.882,0.055] |
| S4 | -0.375 | -1.814^**^ | -0.687 | 0.583 | -1.844^***^ | -0.359 |
|  | [-1.628,0.879] | [-2.990,-0.638] | [-1.918,0.544] | [-0.335,1.501] | [-2.752,-0.937] | [-1.305,0.588] |
| S5 | 0.176 | -0.785 | -0.060 | 0.812 | -0.775 | -0.946 |
|  | [-0.721,1.073] | [-1.678,0.109] | [-0.992,0.873] | [-0.398,2.022] | [-1.983,0.433] | [-2.190,0.298] |
| S6 | -1.288 | 0.268 | -0.864 | -1.399^**^ | -1.987^***^ | -1.332^*^ |
|  | [-3.036,0.459] | [-1.466,2.003] | [-2.615,0.888] | [-2.425,-0.373] | [-3.036,-0.937] | [-2.375,-0.290] |

* Note: * p<0.05, ** p<0.01, *** p<0.001; Confidence Intervals in Brackets below DiD-estimates.
